# District-Level Forecast of Achieving Trachoma Elimination as a Public Health Problem By 2030: An Ensemble Modelling Approach

**DOI:** 10.1093/cid/ciae031

**Published:** 2024-04-25

**Authors:** Ariktha Srivathsan, Amza Abdou, Tawfik Al-Khatib, Sue-Chen Apadinuwe, Mouctar D Badiane, Victor Bucumi, Tina Chisenga, George Kabona, Martin Kabore, Sarjo Kebba Kanyi, Lucienne Bella, Nekoua M’po, Michael Masika, Abdellahi Minnih, Henis Mior Sitoe, Sailesh Mishra, Nicholas Olobio, Fatma Juma Omar, Isaac Phiri, Salimato Sanha, Fikre Seife, Shekhar Sharma, Rabebe Tekeraoi, Lamine Traore, Titus Watitu, Yak Yak Bol, Anna Borlase, Michael S Deiner, Kristen K Renneker, P J Hooper, Paul M Emerson, Andreia Vasconcelos, Benjamin F Arnold, Travis C Porco, T Déirdre Hollingsworth, Thomas M Lietman, Seth Blumberg

**Affiliations:** Francis I. Proctor Foundation, University of California, San Francisco, California, USA; Programme National de Santé Oculaire, Ministère De La Santé Publique, Niamey, Niger; Prevention of Blindness Program, Ministry of Public Health & Population, Sana'a, Yemen; Ministry of Health and Medical Services, Denig, Republic of Nauru; Programme National de Promotion de La Santé Oculaire, Ministère de la Santé et de L'Action sociale, Dakar, Sénégal; Département En Charge des Maladies Tropicales Négligées, Ministère De La Santé Publique Et De La Lutte Contre Le Sida, Bujumbura, Burundi; Ministry of Health Public Health Department, Lusaka, Zambia; Neglected Tropical Disease Control Program, Ministry of Health and Social Welfare, Dar Es Salaam, United Republic of Tanzania; Programme national de lutte contre les maladies tropicales négligées, Ministère de la santé et de l'hygiène publique, Ouagadougou, Burkina Faso; The National Eye Health Programme, Ministry of Health and Social Welfare, Banjul, Kanifing, The Gambia; Programme National De Lutte Contre La Cécité, Ministère De La Santé Publique, Yaoundé, Cameroon; Programme National De Lutte Contre Les Maladies Transmissibles, Ministère De La Santé, Cotonou, Benin; Department of Clinical Services, Ministry of Health, Lilongwe, Malawi; Département Des Maladies Transmissibles, Ministère De La Santé Nouakchott, Nouakchott, Mauritania; Direcção Nacional De Saúde Pública Ministerio Da Saude, Maputo, Mozambique; Nepal Netra Jyoti Sangh, Kathmandu, Nepal; National Trachoma Elimination Programme, Federal Ministry of Health, Abuja, Nigeria; Zanzibar Ministry of Health, Zanzibar Town, Zanzibar; Department of Epidemiology and Disease Control, Ministry of Health & Child Welfare, Harare, Zimbabwe; Programa Nacional De Saúde De Visão, Minsap, Bissau, Guinea-Bissau; Federal Ministry of Health, Addis Ababa, Ethiopia; Nepal Netra Jyoti Sangh, Kathmandu, Nepal; Eye Department, Ministry of Health and Medical Services, South Tarawa, Kiribati; Programme National de la Santé Oculaire, Ministère de la Santé, Bamako, Mali; Ministry of Health, Nairobi, Kenya; Neglected Tropical Diseases Programme, Ministry of Health, Juba, South Sudan; Department of Biology, University of Oxford, Oxford, United Kingdom; Francis I. Proctor Foundation, University of California, San Francisco, California, USA; International Trachoma Initiative, The Task Force for Global Health, Decatur, Georgia, USA; International Trachoma Initiative, The Task Force for Global Health, Decatur, Georgia, USA; International Trachoma Initiative, The Task Force for Global Health, Decatur, Georgia, USA; Big Data Institute, Li Ka Shing Centre for Health Information and Discovery, University of Oxford, Oxford, United Kingdom; Francis I. Proctor Foundation, University of California, San Francisco, California, USA; Francis I. Proctor Foundation, University of California, San Francisco, California, USA; Big Data Institute, Li Ka Shing Centre for Health Information and Discovery, University of Oxford, Oxford, United Kingdom; Francis I. Proctor Foundation, University of California, San Francisco, California, USA; Francis I. Proctor Foundation, University of California, San Francisco, California, USA; Department of Medicine, University of California, San Francisco, California, USA

## Abstract

Assessing the feasibility of 2030 as a target date for global elimination of trachoma, and identification of districts that may require enhanced treatment to meet World Health Organization (WHO) elimination criteria by this date are key challenges in operational planning for trachoma programmes. Here we address these challenges by prospectively evaluating forecasting models of trachomatous inflammation–follicular (TF) prevalence, leveraging ensemble-based approaches. Seven candidate probabilistic models were developed to forecast district-wise TF prevalence in 11 760 districts, trained using district-level data on the population prevalence of TF in children aged 1–9 years from 2004 to 2022. Geographical location, history of mass drug administration treatment, and previously measured prevalence data were included in these models as key predictors. The best-performing models were included in an ensemble, using weights derived from their relative likelihood scores. To incorporate the inherent stochasticity of disease transmission and challenges of population-level surveillance, we forecasted probability distributions for the TF prevalence in each geographic district, rather than predicting a single value. Based on our probabilistic forecasts, 1.46% (95% confidence interval [CI]: 1.43–1.48%) of all districts in trachoma-endemic countries, equivalent to 172 districts, will exceed the 5% TF control threshold in 2030 with the current interventions. Global elimination of trachoma as a public health problem by 2030 may require enhanced intervention and/or surveillance of high-risk districts.

Trachoma, an ocular disease caused by *Chlamydia trachomatis*, is the leading cause of infection blindness in the world [1]. The WHO recommended the SAFE strategy for trachoma control, which includes surgery to treat trachomatous trichiasis, antibiotics to clear active infection, facial cleanliness, and environmental improvement [2,3]. Although this strategy has empirically been shown to be effective in reducing disease burden [4], interventions may need to be intensified in areas with persistent infection [5].

The WHO Alliance for the Global Elimination of Trachoma by the year 2020 (GET2020) was launched in 1996 [6]. The alliance supports and collaborates with the WHO to support Ministries of Health and Non-Governmental Development Organizations’ programmatic efforts toward eliminating trachoma as a public health problem by 2020 [7]. Elimination of trachoma as a public health problem (EPHP) requires a prevalence of trachomatous inflammation–follicular in children aged 1–9 years (TF_1–9_) of <5%, sustained for at least two years in the absence of ongoing antibiotic mass treatment, in each formerly endemic district [8, 9].

However, by 2020 only 15% of endemic countries had met the EPHP target, and thus global EPHP was not attained. The road map for neglected tropical diseases 2021–2030, endorsed by the World Health Assembly in 2020, set 2030 as the new target date for global EPHP [10]. With 22% of endemic countries having achieved the target as of 2022 [3], it is still unclear if global elimination by 2030 is attainable. Forecasting the prevalence of trachoma in endemic countries using statistically robust methods represents a useful tool for ascertaining the feasibility of the 2030 deadline and identifying areas that might benefit from enhanced control activities.

Both mechanistic and statistical models have been used to forecast the prevalence of infectious diseases, including trachoma. Most commonly, mechanistic models such as the susceptible, infected, susceptible (SIS) model structure have been used in trachoma modeling [11–16]. However, forecasts generated by mechanistic models do not necessarily perform better than forecasts generated by purely statistical models [17]. There has been a recent shift towards probabilistic forecasts that provide both a predicted outcome and the uncertainty of the prediction [18]. Additionally, several studies have demonstrated that ensemble models used to forecast disease prevalence may perform better than their constituent models [19–21].

In the present study, we use available survey data to build an ensemble of probabilistic models to forecast the prevalence of TF_1–9_ in 2030. In addition to evaluating whether 2030 is a reasonable goal for global EPHP, the ensemble can be used to forecast TF_1–9_ in regions without prior surveys and identify regions where enhanced intervention may be required to meet EPHP by 2030.

## METHODS

### Data

Implementation unit (IU) level TF_1–9_ and trachomatous trichiasis (TT) prevalence surveys and mass drug administration (MDA) distribution data were accessed through the GET2020 database for each IU. The GET2020 database is maintained by the International Trachoma Initiative (ITI) in partnership with WHO. An IU is defined as an administrative unit at which trachoma implementation activities, such as surveys and mass drug administration take place, and typically contains 100 000–250 000 people [9]. Additionally, IU-level geographic information was provided by ITI.

**Figure 1.**
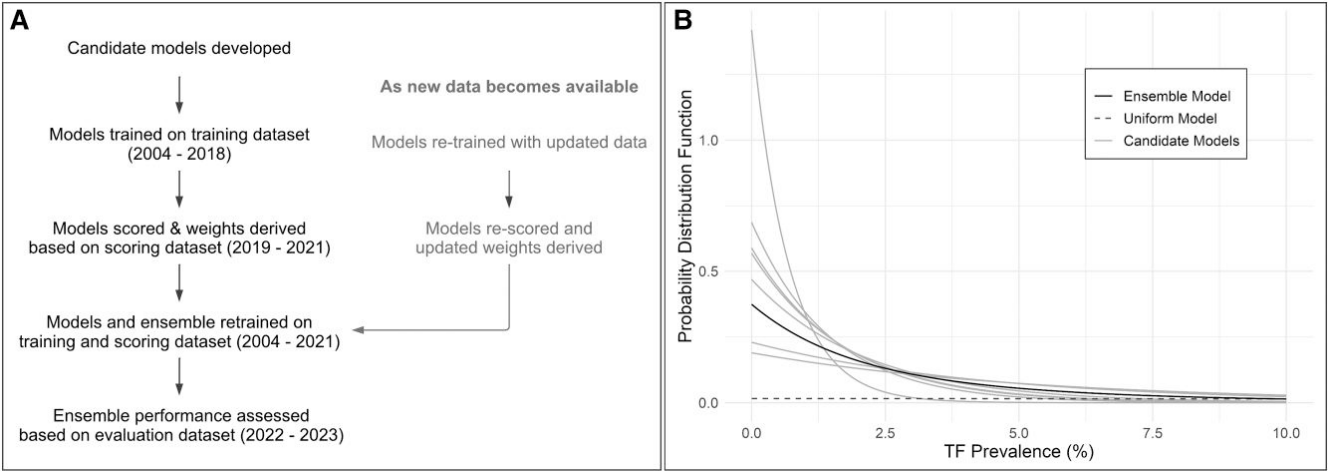
*A*, A schematic of the methodology used to derive IU-wise probabilistic forecasts of trachoma prevalence. The method evaluates the accuracy of a suite of candidate models and then combines the highest performing ones to form an ensemble forecast. *B*, Sample forecast for a sample district for 2030 is represented as the probability distribution function defined over the range of 0%–100% TF_1–9_. The candidate models (*light gray lines*), that perform better than the uniform model (dotted gray line), are included in the ensemble model (*black line*). By forecasting the distribution of observed TF values, various metrics can be calculated, such as the probability of achieving EPHP by 2030. Abbreviations: EPHP, elimination of trachoma as a public health problem; IU, implementation unit; TF, trachomatous inflammation–follicular.

The available data were divided longitudinally into 3 parts: a training data set (2004–2018), a scoring data set (2019–2021), and an evaluation data set (2022–2023). All the candidate models were trained on the training data set. The scoring and subsequent assignment of weights for our ensemble models was based on the scoring data set. The performance of the generated ensembles and the candidate models was assessed based on the evaluation data set (Figure 1*A* ).

### Candidate Models

Seven candidate models were considered for inclusion in the ensemble, each of which is an IU-specific probabilistic model (Figure 1*B* ). That is, each candidate model predicts a probability that TF has a range of specific values for each district for each year. Forecasts were provided as probability distribution functions (PDFs) for each year of 2019–2030.

We use 2 families of distribution to describe the observed frequency distributions. First, the truncated exponential distribution was used to model TF_1–9_ observation as a function of the most likely value for TF_1–9_. This distribution was chosen as it is the maximum entropy distribution given the prevalence range of 0%–100%, and the mean of the distribution [22]. Additionally, the exponential distribution is consistent with theoretical models of disease elimination [14, 23, 24] and observed trends [25, 26].

Second, a uniform distribution over the range of 0%–100% TF_1–9_ prevalence is used as a simple baseline model to assess the predictive accuracy of candidate models. Candidate models were included in the ensemble if they performed better than this uniform model. This, along with the ensemble weighting described below, provides a quantitative evaluation that models maintain predictive ability.

Spatial aggregation-based models:

There are many different spatial scales at which we could choose to aggregate the data to give a frequency distribution of TF prevalence—for example districts within a country, regions or the whole continent. For each geographic aggregation, a maximum likelihood fit is determined by assuming that the TF_1–9_ values for each year follow an exponential distribution that is truncated to allow TF_1–9_ values of 0% to 100%. The mean of the underlying (non-truncated) distribution is modeled to follow an exponential decay as a function of year.

- Country-level aggregation: For the country model, the unit is a country, except for countries with <50 surveys in the training data. All countries that did not meet this threshold were considered one cluster.
- Region-level aggregation: Analogous to country-level aggregation, the unit here is within-country regions, as defined within the GET 2020 database. Regions with <30 surveys in the training data\were aggregated at the country level. Whenever aggregation on a country-level also failed to reach the threshold of 30 surveys, the associated surveys were included in one catch-all cluster.
- Global model: For the global model, no geographic aggregation is performed. This generates an IU-agnostic forecast that has no spatial variation but varies over time.

Regression based models:

As before, the model for the PDF of the observed TF_1–9_ values is a truncated exponential distribution. However, for these models, the mean of the distribution is obtained by training a linear regression model for the log of TF_1–9_ prevalence.

- Fixed effects model: Previous TF_1–9_ prevalence, regional average of TF_1–9_, Year (as proxy for baseline trend), and the number of rounds of MDA previously delivered were used to train a district-specific model for TF_1–9_ on the logarithmic scale. Final variable selection for the candidate model was achieved via stepwise regression using Bayes Information Criterion (BIC).
- Mixed effects model: The same method as for the fixed-effects model was implemented with an additional random intercept term for each country.
- Geospatial model: A geostatistical model [27] using a Matern spatial covariance function and year as the only other covariate was used to model the district-wise TF_1–9_ prevalence on the logarithmic scale.

Naive Exponential model: The average TF_1–9_ prevalence of all surveys in the last year of the training data was used as the mean of an exponential fit. The resulting model is a forecasting model that is the same for each district and does not change with time.

### Scoring

To evaluate probabilistic forecasts, we utilized 2 proper scores, the logarithmic score (LogS) and continuous ranked probability score (CRPS) [28]. Proper scores incentivize honest forecasting—that is, the best score is obtained by reporting the true distribution [29]. When scoring a data set for a specific model, the individual scores for each TF_1–9_ prevalence in the dataset are summed. Lower scores represent better forecasts.

Sensitivity analysis was performed to look for variation in scores based on which score is used and for the years included in the scoring dataset.

### Ensemble Model

Ensembles were created as weighted averages of candidate models. The weight, *w*, that a candidate model, *c_i_,* contributes to the ensemble is related to the likelihood of the data for each candidate model, given by the exponential of the overall LogS score. The weights were tempered by a constant, *k*, to limit the over-contribution of any one model to the ensemble.


$$w(c_{i})=\frac{e^{\frac{-\Sigma LogS(c_{i})}{k}}}{\sum_{i}e^{\frac{-\Sigma LogS(c_{i})}{k}}}$$

Ensembles were generated using 4 values of *k* (1, 10, 100, 1000).

### Model Outputs

The forecast of each candidate model and the resulting ensembles were represented as a probability distribution for each IU for each year. This format captures the prediction and the uncertainty of the forecast. Different summary metrics can then be constructed for each district, such as the probability that a district will reach the TF_1–9_ < 5% target for EPHP by 2030.

All analyses were performed using R Statistical Software [30].

## RESULTS

### Data

Trachoma survey data and MDA distribution data was retrieved from the GET2020 database as of February 2023, containing data from 1985 to 2023. After excluding any data points that were incomplete for TF_1–9_ prevalence, survey year or IU, and excluding data prior to 2004 when TF_1–9_ diagnosis was less uniform, 5898 surveys in 2597 IUs across 25 countries were included in the study (Table 1). Of these surveys, 74% were included in the training data set (2004–2018), 19% in the scoring data set (2019–2021), and 7% in the evaluation data set (2022–2023). The distribution of TF_1–9_ prevalence over the years shows an increasing density toward the left, that is, an increasing probability of TF_1–9_ < 5% over time (Supplementary Figure 1).

**Table 1.** Characteristics of Included Data

Number of surveys	5898
Years	2004–2023
TF Prevalence: Mean % (range)	9.1 (0–80.1)
TF Prevalence: Median % (IQR)	4.1 (1.2–11.7)
TT Prevalence: Mean % (range)	0.9 (0–23.5)
TT Prevalence: Median % (IQR)	0.3 (0.1–1.0)
Countries	25
Total number of implementation units	2597
Rounds of MDA, mean (range)	2.3 (0–16)

Abbreviations: IQR, interquartile range; MDA, Mass Drug Administration; TF, Trachomatous inflammation—follicular; TT, Trachomatous trichiasis.

### Candidate Model Evaluation

All 7 candidate models described in the methods section were eligible to be included in the ensembles because they all outperformed the uniform model. Excluded models that performed worse than the uniform model included those with a Gaussian-shaped probability density function. The poorly performing models presumably did not represent uncertainty in TF_1–9_ measurement adequately. The ranks based on both scoring rules were fairly consistent, with regression-based models outperforming the aggregation models. The fixed effects model was the best performing (lowest scoring) model based on both scoring rules. Additionally, while the models performed consistently on all years in the scoring data set based on LogS, the models consistently performed worse in 2019 based on CRPS (result not shown).

### Ensembles Models and Their Evaluation

**Figure 2.**
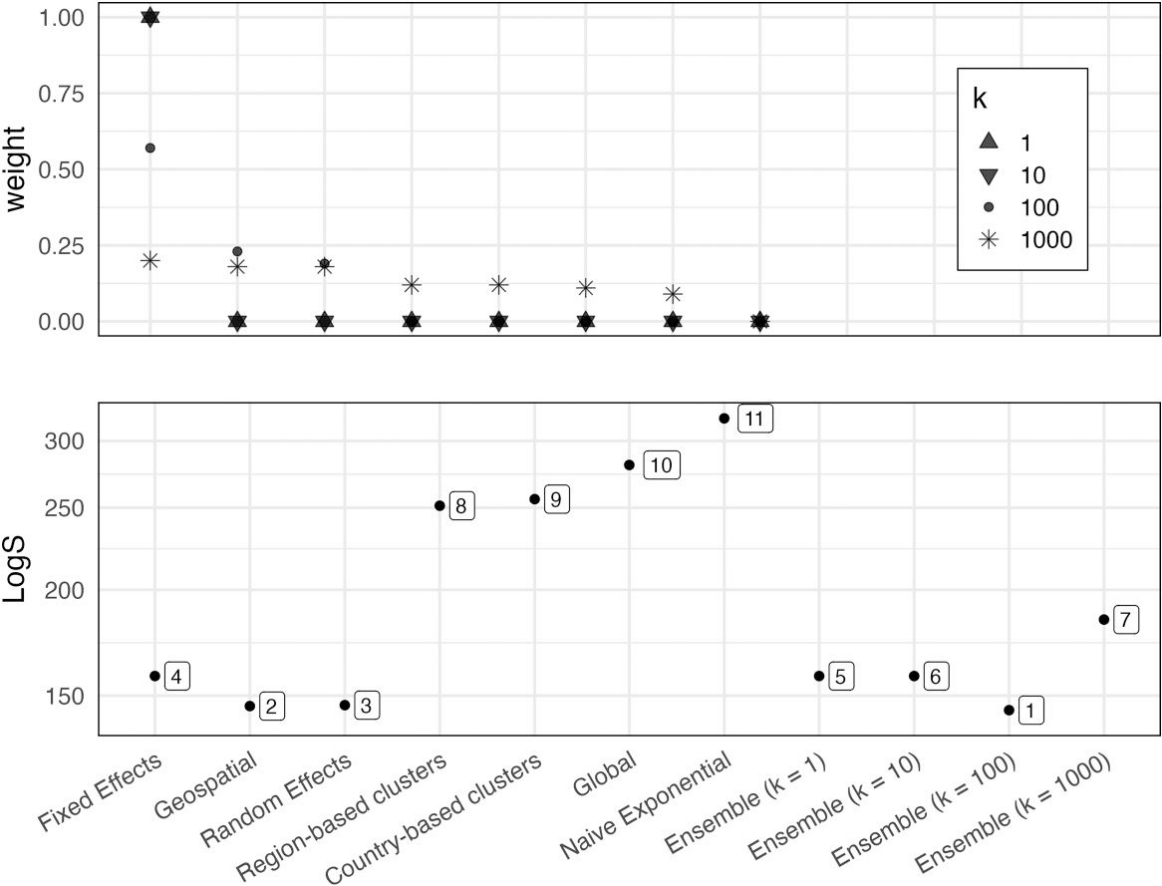
(*Top panel*): Weight of each candidate model in the ensembles by tempering constant, *k*. Higher weights indicate more influence in the ensemble model. Higher levels of tempering reduce the influence of any one model by reducing the scale of the scores that candidate models can achieve. (*Bottom panel*): Cumulative log score of the ensembles and 7 candidate models based on the evaluation data set (2022–2023). Lower scores represent better agreement with data. The numbers indicate the overall rank of each model.

When determining the mix of candidate models used in our ensemble models, tempering with low values of k effectively excluded all but the fixed effects model. Increasing the value of k (k = 1000) made the weights more uniform. This decreased the maximum contribution of a single model from 100% to 20% (Figure 2*A* ).

Based on both scoring rules, the ensemble with *k* = 100 was the best performing model on the evaluation dataset. Consistent with the use of ensemble models in other applications, the ensemble model performed better than its constituent models. Scores for the candidate models on the evaluation data set were consistent with that on the scoring data set, with the regression-based models outperforming the clustering-based models (Figure 2*B* ).

### Global EPHP 2030

**Figure 3:**
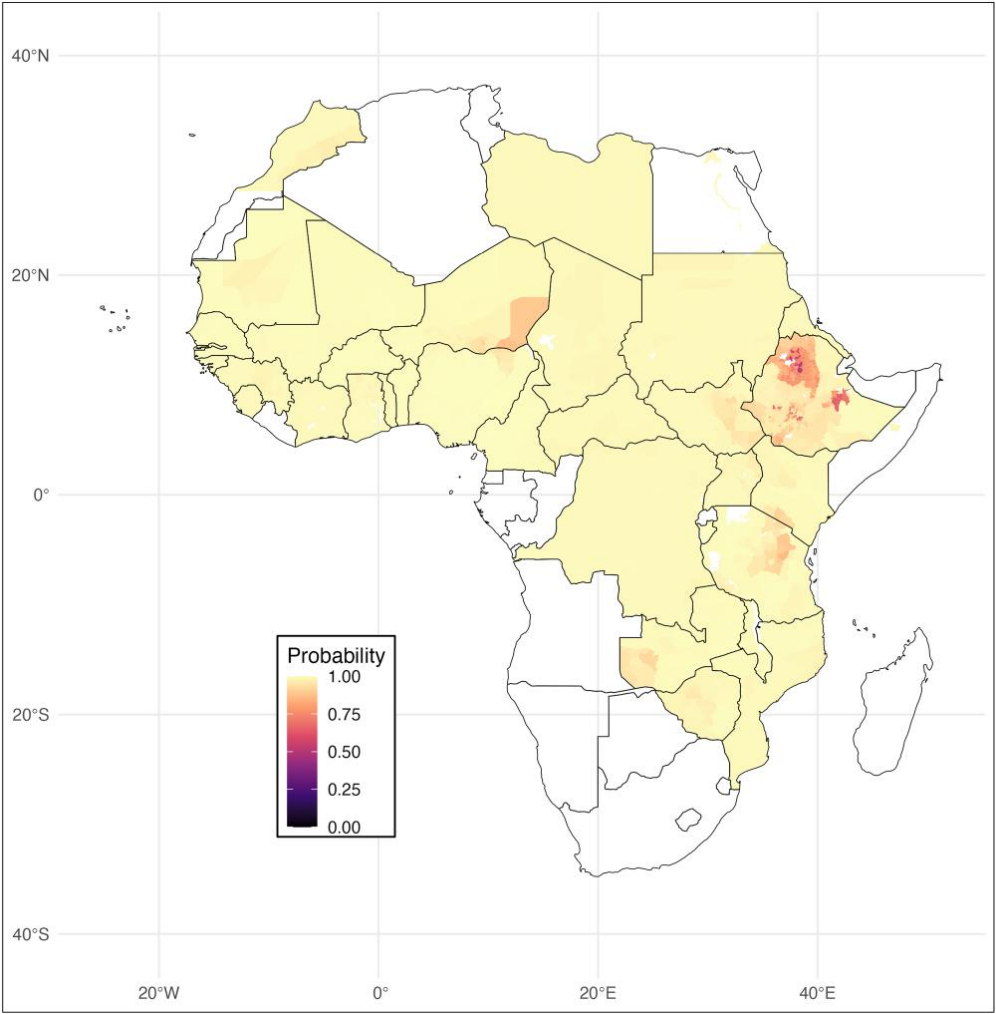
Map of Africa depicting the probability that TF will be <5% in 2030, which would satisfy the WHO requirement for elimination of trachoma as a public health problem. Results are based on the ensemble model with k = 100. Darker colors depict a lower probability of TF being controlled by the target date. Yellow areas of the map depict areas with a high probability of control. Abbreviations: TF, trachomatous inflammation–follicular; WHO, World Health Organization.

Based on the forecast from the ensemble with *k* = 100, it is expected that with current treatment, 1.46% [1.43%–1.48%] (172/11 760) IUs in previously endemic countries will not attain elimination as a public health problem by 2030 (Figure 3). Thus, global EPHP by 2030 will likely require enhanced intervention and/or surveillance in high-risk IUs.

## DISCUSSION

According to our forecast, approximately 172 IUs will not attain EPHP by 2030 under current trends. Enhanced interventions, such as biannual MDA, or novel interventions, such as a vaccine, could be particularly useful in the IUs not expected to reach control under current trends. Such interventions could be modeled to assess their adequacy to help these IUs meet the target. Additionally, using map information and data from other IUs in the region, we can predict TF_1–9_ in areas where no surveys have taken place. This insight could be leveraged to plan targeted future surveys in those regions.

Because it informs optimal distribution of treatment and other interventions, the value of district-specific forecasting in modeling the prevalence of TF_1–9_ may be most pronounced as we approach global EPHP. Meanwhile, forecasting is generally more accurate in the short term and enhanced interventions could improve outcomes [31, 32]. This warrants mid-term goals that can be used to measure progress and revisiting the 2030 forecasts periodically with updated data.

A key strength of our modeling approach is its dynamic nature—each model can be re-trained as more survey data becomes available, making updated forecasts responsive to global and local changes in trachoma prevalence and trends. In addition to being able to include new data, additional models can also be added to the ensemble to improve the accuracy and certainty of the forecast. However, due to the statistical nature of the forecasting method, mechanistic conclusions such as intervention efficacy or the secular trend cannot be drawn directly from the ensemble model.

The ensemble outperformed its constituent models on the evaluation dataset, based on both scoring rules used. This effect was more pronounced on CRPS, indicating that the ensemble reduces the forecasting error. Additionally, tempering the weights that each candidate model contributes to the ensemble improved the performance as compared to a non-tempered (*k* = 1) model. This indicates that the best performing candidate model may vary each year, potentially attributable to a non-random sample of IUs being surveyed each year.

IU-specific models performed better than the IU-agnostic models (global and naïve exponential models) on both the scoring and evaluation datasets, indicating IU-wise heterogeneity. Additionally, the geospatial model was the most consistently performing model across years in the scoring dataset, suggesting that smoothing over neighboring IUs adjusts for some of the stochasticity of TF_1–9_.

In the present study we chose TF_1–9_ as the prevalence marker for forecasting due to its integral role in WHO's definition of elimination of trachoma as a public health problem. However, TF_1–9_ has a low sensitivity for active infection because the post-infectious inflammation can take months to resolve [33]. Additionally, TF_1–9_ factors such as inter-rater variability make it a noisy measure. This adds to the complexity of making public health decisions based on TF_1–9_ alone and motivates an assessment of bias in TF_1–9_ measurements. The incorporation of other prevalence measures, such as Trachomatous Trichiasis and Anti-Pgp3 serology might alleviate some challenges in forecasting IU-specific TF_1–9_ and the relationship to ongoing transmission.

The models included a host of predictors, both explicitly and implicitly. Factors such as spatial variability [34], MDA interventions [35, 36], and secular (ie, not dependent on MDA) decline of trachoma prevalence [37] are included explicitly as covariates in various candidate models. On the other hand, the specific impact of interventions such as facial cleanliness, and environmental improvement (F&E) [38] are included by proxy as these interventions are primarily implemented together with MDA. Additionally, F&E are believed to be a contributor to the secular trend.

Each candidate model has its own limitations—such as the global model not accounting for geospatial trends, the non-independence of observations for regression-based models, and the ad-hoc clustering of IUs in the aggregation models. However, the creation of ensemble models draws on the strengths of each of the candidate models by weighting them based on the accuracy of their predictions.

Although WHO's 2030 goal of global EPHP may seem arbitrary, current results suggest that it is achievable in most regions. Enhanced intervention may be required to meet the goal in certain regions, and district-wise forecasts can be used to target IUs that might benefit the most from such interventions.

## Supplementary Data

Supplementary materials are available at *Clinical Infectious Diseases* online. Consisting of data provided by the authors to benefit the reader, the posted materials are not copyedited and are the sole responsibility of the authors, so questions or comments should be addressed to the corresponding author.

## Supplementary Material

ciae031_Supplementary_Data

## Notes

***Acknowledgments.*** The authors express their gratitude to all partners involved in trachoma surveillance, who have gathered the data serving as the foundation for this article. The collection of this data necessitated the examination of millions of individuals by numerous field teams, trainers, supervisors, statisticians, epidemiologists, data analysts, project managers, and countless others. They extend their sincere appreciation to all these individuals for their invaluable contributions in generating the GET2020 database.

***Financial Support.*** The work presented in this article is funded by the National Eye Institute (NEI) at the National Institutes of Health (NIH) (grant number R01EY025350), NTD Modelling Consortium funded by the Bill & Melinda Gates Foundation (grant number INV-030046), Children's Investment Foundation Fund, and an Unrestricted Grant from Research to Prevent Blindness.

***Data availability.*** The data used in this analysis is a part of the GET 2020 database, maintained by the International Trachoma Initiative in partnership with WHO. The data belong to each individual country included. Data on TF_1–9_ prevalence category and post-2010 mass drug administration are publicly available on the Global Atlas of Trachoma (https://www.trachomaatlas.org/ ). Any request for study data not available on the Global Atlas of Trachoma must be made directly with countries and will be assessed on a case-by-case basis at each country's discretion.

The R scripts used for this analysis are available at: https://github.com/ariktha/tf-forecast-ensemble.git .

***Supplement sponsorship.*** This article appears as part of the supplement “New Tools and Nuanced Interventions to Accelerate Achievement of 2030 Roadmap for Neglected Tropical Diseases,” sponsored by funding of Professor T. Déirdre Hollingsworth's research by the Li Ka Shing Foundation at the Big Data Institute, Li Ka Shing Centre for Health Information and Discovery, University of Oxford; and funding of the NTD Modelling Consortium by the Bill & Melinda Gates Foundation (INV-030046).

## References

[1] Flaxman SR, Bourne RRA, Resnikoff S, et al Global causes of blindness and distance vision impairment 1990–2020: a systematic review and meta-analysis. Lancet Glob Health 2017; 5:e1221–34. 29032195 10.1016/S2214-109X(17)30393-5

[2] Kuper H, Solomon AW, Buchan J, Zondervan M, Foster A, Mabey D. A critical review of the SAFE strategy for the prevention of blinding trachoma. Lancet Infect Dis 2003; 3:372–81. 12781509 10.1016/s1473-3099(03)00659-5

[3] World Health Organization . Trachoma Fact Sheet. Available at: https://www.who.int/news-room/fact-sheets/detail/trachoma . Accessed 13 June 2023.

[4] WHO Alliance for the Global Elimination of Trachoma by 2020: progress report, 2019. Available at: https://www.who.int/publications-detail-redirect/who-wer9530 . Accessed 31 December 2023.

[5] Lavett DK, Lansingh VC, Carter MJ, Eckert KA, Silva JC. Will the SAFE strategy be sufficient to eliminate trachoma by 2020? Puzzlements and possible solutions. ScientificWorldJournal 2013; 2013:648106. 23766701 10.1155/2013/648106 PMC3671555

[6] WHO Alliance for the Global Elimination of Trachoma . Meeting (1^st^: 1997: Geneva S, Deafness WP for the P of B and. Report of the first meeting of the WHO Alliance for the Global Elimination of Trachoma, Geneva, Switzerland, 30 June to 1 July 1997. World Health Organization, 1997. Available at: https://apps.who.int/iris/handle/10665/66178 . Accessed 28 March 2023.

[7] WHA51.11 . Global elimination of blinding trachoma. 1998; Available at: https://apps.who.int/iris/bitstream/handle/10665/79806/ear11.pdf . Accessed 28 March 2023.

[8] Meeting for the development of guidelines for assessment of the elimination of blinding trachoma. Geneva, Switzerland: World Health Organization, Prevention of Blindness & Deafness, **2001**.

[9] World Health Organization. Validation of elimination of trachoma as a public health problem . Available at: https://apps.who.int/iris/handle/10665/208901 . Accessed 28 March 2023.

[10] Ending the neglect to attain the sustainable development goals: a road map for neglected tropical diseases 2021–2030. Geneva: World Health Organization, 2020. Available at: https://www.who.int/publications-detail-redirect/9789240010352 . Accessed 28 March 2023.

[11] Lietman TM, Gebre T, Ayele B, et al The epidemiological dynamics of infectious trachoma may facilitate elimination. Epidemics 2011; 3:119–24. 21624783 10.1016/j.epidem.2011.03.004 PMC3869790

[12] Liu F, Porco TC, Ray KJ, et al Assessment of transmission in trachoma programs over time suggests no short-term loss of immunity. PLoS Negl Trop Dis 2013; 7:e2303. 23875038 10.1371/journal.pntd.0002303 PMC3708821

[13] Gambhir M, Basáñez M-G, Burton MJ, et al The development of an age-structured model for trachoma transmission dynamics, pathogenesis and control. PLoS Negl Trop Dis 2009; 3:e462. 19529762 10.1371/journal.pntd.0000462 PMC2691478

[14] Ray KJ, Porco TC, Hong KC, et al A rationale for continuing mass antibiotic distributions for trachoma. BMC Infect Dis 2007; 7:91. 17683646 10.1186/1471-2334-7-91 PMC1988814

[15] Ray KJ, Lietman TM, Porco TC, et al When can antibiotic treatments for trachoma be discontinued? Graduating communities in three African countries. PLoS Negl Trop Dis 2009; 3:e458. 19529761 10.1371/journal.pntd.0000458 PMC2690652

[16] Borlase A, Blumberg S, Callahan EK, et al Modelling trachoma post-2020: opportunities for mitigating the impact of COVID-19 and accelerating progress towards elimination. Trans R Soc Trop Med Hyg 2021; 115:213–21. 33596317 10.1093/trstmh/traa171 PMC7928577

[17] Lietman TM, Pinsent A, Liu F, Deiner M, Hollingsworth TD, Porco TC. Models of trachoma transmission and their policy implications: from control to elimination. Clin Infect Dis 2018; 66:S275–80. 29860288 10.1093/cid/ciy004 PMC5982784

[18] Pinsent A, Liu F, Deiner M, et al Probabilistic forecasts of trachoma transmission at the district level: a statistical model comparison. Epidemics 2017; 18:48–55. 28279456 10.1016/j.epidem.2017.01.007 PMC5340843

[19] Oidtman RJ, Omodei E, Kraemer MUG, et al Trade-offs between individual and ensemble forecasts of an emerging infectious disease. Nat Commun 2021; 12:5379. 34508077 10.1038/s41467-021-25695-0 PMC8433472

[20] Bannick MS, McGaughey M, Flaxman AD. Ensemble modelling in descriptive epidemiology: burden of disease estimation. Int J Epidemiol 2020; 49:2065–73. 10.1093/ije/dyz223 31722368

[21] Shashvat K, Basu R, Bhondekar AP, Kaur A. A weighted ensemble model for prediction of infectious diseases. Curr Pharm Biotechnol. 2019; 20:674–8. 31203798 10.2174/1389201020666190612160631

[22] Conrad K . Probability distributions and maximum entropy. Entropy 2004; 6:10.

[23] Nåsell I . On the quasi-stationary distribution of the stochastic logistic epidemic. Math Biosci 1999; 156:21–40. 10204386 10.1016/s0025-5564(98)10059-7

[24] Lietman TM, Gebre T, Abdou A, et al The distribution of the prevalence of ocular chlamydial infection in communities where trachoma is disappearing. Epidemics 2015; 11:85–91. 25979286 10.1016/j.epidem.2015.03.003 PMC4986606

[25] Rahman SA, West SK, Mkocha H, et al The distribution of ocular Chlamydia prevalence across Tanzanian communities where trachoma is declining. PLoS Negl Trop Dis 2015; 9:e0003682. 25815466 10.1371/journal.pntd.0003682 PMC4376383

[26] Hiep NX, Ngondi JM, Anh VT, et al Trachoma in Viet Nam: results of 11 surveillance surveys conducted with the Global Trachoma Mapping Project. Ophthalmic Epidemiol 2018; 25:93–102. 30806534 10.1080/09286586.2018.1477964 PMC6444206

[27] Rousset F, Ferdy J-B. Testing environmental and genetic effects in the presence of spatial autocorrelation. Ecography 2014; 37:781–90.

[28] Hersbach H . Decomposition of the continuous ranked probability score for ensemble prediction systems. Weather Forecasting 2000; 15:559–70.

[29] Gneiting T, Raftery AE. Strictly proper scoring rules, prediction, and estimation. J Am Stat Assoc 2007; 102:359–78.

[30] R Core Team . R: A Language and Environment for Statistical Computing. R Foundation for Statistical Computing, Vienna, Austria 2022; https://www.R-project.org/ Accessed 28 March 2023

[31] Pei S, Cane MA, Shaman J. Predictability in process-based ensemble forecast of influenza. PLoS Comput Biol 2019; 15:e1006783. 30817754 10.1371/journal.pcbi.1006783 PMC6394909

[32] Scarpino SV, Petri G. On the predictability of infectious disease outbreaks. Nat Commun 2019; 10:898. 30796206 10.1038/s41467-019-08616-0 PMC6385200

[33] Solomon AW, Burton MJ, Gower EW, et al Trachoma. Nat Rev Dis Primers 2022; 8:32. 35618795 10.1038/s41572-022-00359-5

[34] Amoah B, Fronterre C, Johnson O, et al Model-based geostatistics enables more precise estimates of neglected tropical-disease prevalence in elimination settings: mapping trachoma prevalence in Ethiopia. Int J Epidemiol 2022; 51:468–78. 34791259 10.1093/ije/dyab227 PMC9082807

[35] Bailey RL, Arullendran P, Whittle HC, Mabey DC. Randomised controlled trial of single-dose azithromycin in treatment of trachoma. Lancet 1993; 342:453–6. 8102427 10.1016/0140-6736(93)91591-9

[36] Schachter J, West SK, Mabey D, et al Azithromycin in control of trachoma. Lancet 1999; 354:630–5. 10466664 10.1016/S0140-6736(98)12387-5

[37] Chidambaram JD, Bird M, Schiedler V, et al Trachoma decline and widespread use of antimicrobial drugs. Emerg Infect Dis 2004; 10:1895–9. 15550197 10.3201/eid1011.040476 PMC3329000

[38] Stocks ME, Ogden S, Haddad D, Addiss DG, McGuire C, Freeman MC. Effect of water, sanitation, and hygiene on the prevention of trachoma: a systematic review and meta-analysis. PLoS Medicine 2014; 11:e1001605. 24586120 10.1371/journal.pmed.1001605 PMC3934994

