# Supplementary material for: District-Level Forecast of Achieving Trachoma Elimination as a Public Health Problem By 2030: An Ensemble Modelling Approach
Source: Clin Infect Dis. 2024 Apr 25;78(Suppl 2):S101–7. doi: 10.1093/cid/ciae031 (PMC11045026; doi:10.1093/cid/ciae031)
Supplement: ciae031_Supplementary_Data [file ciae031_supplementary_data.docx]

**Supplementary Figures**


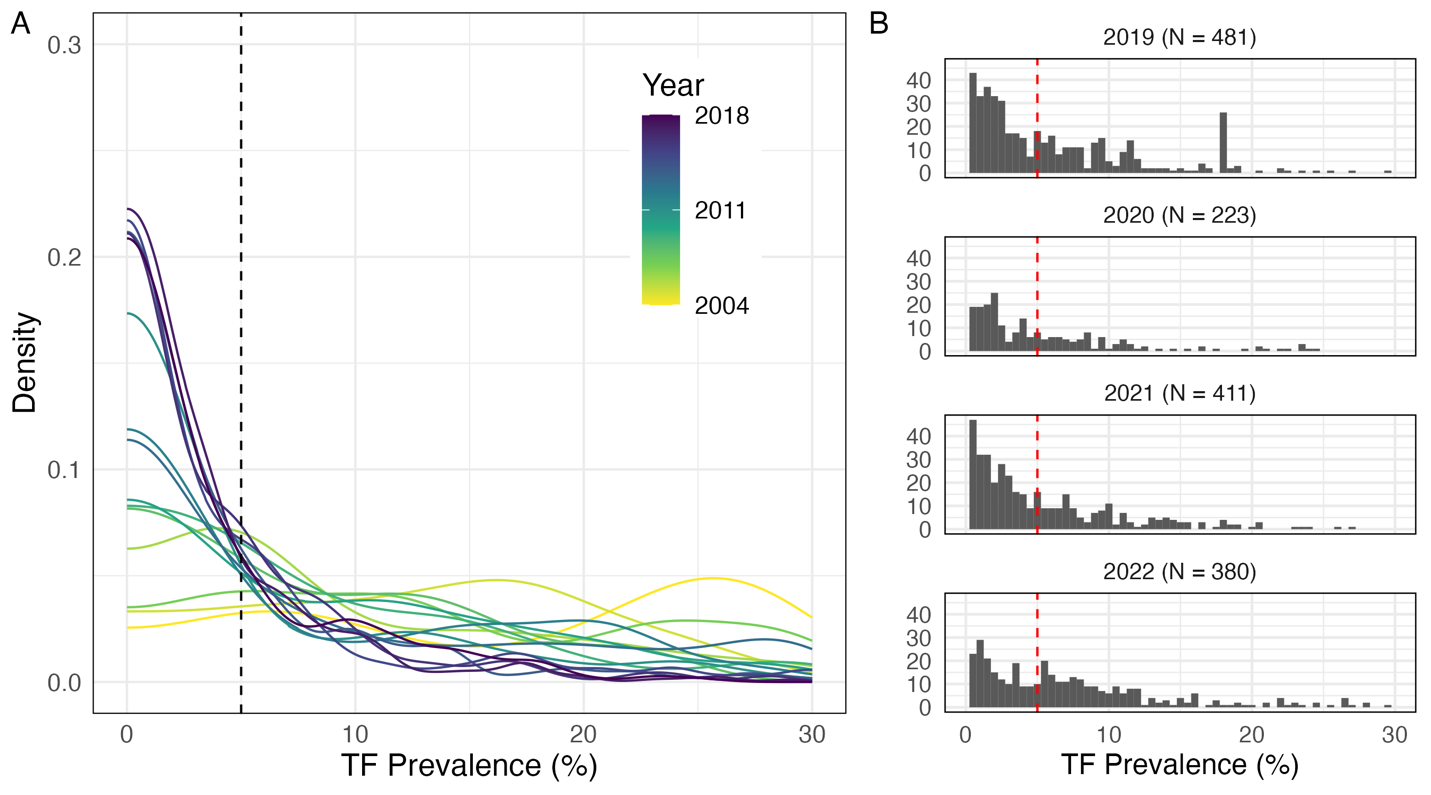


Supplementary Figure 1: Observed prevalence of TF_1-9_: Panel A depicts a density plot of observed TF_1-9_ prevalence by year of data in the training dataset (2004–2018), with darker colors representing more recent years. Panel B depicts histograms of observed TF_1-9_ prevalence by year in the scoring dataset (2019–2022). The dashed vertical line in all plots represents the 5% TF_1-9_ prevalence target required to achieve EPHP. Overall, the density shifts leftward with time, indicating progress towards EPHP. However, many districts are still above target.


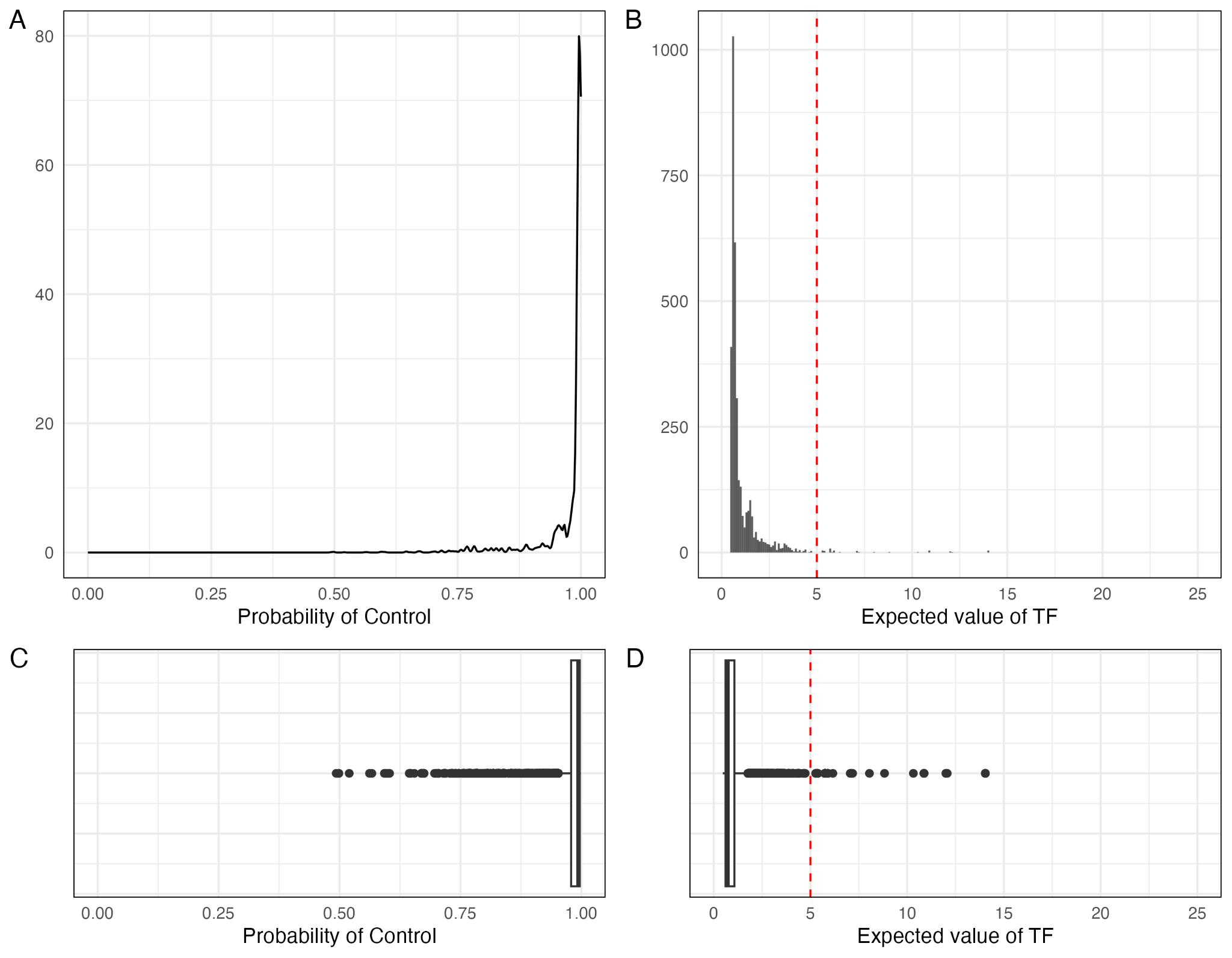


Supplementary Figure 2: Results from the ensemble forecast (k = 100) for 11760 districts for 2030

Panels A and C are a density plot and boxplot respectively of the distribution of the probability of control. Panels B and D are a density plot and boxplot respectively of the distribution of the expected value of TF. The vertical dashed line depicts the 5% threshold of control.
